# Impact of a High-Protein Diet Associated with Probiotics on Lipid Profile, Oxidative Stress, and Mitochondrial Activity in Adipose and Muscle Tissues of Wistar Rats

**DOI:** 10.3390/nu18183070

**Published:** 2026-09-20

**Authors:** Luiza Ferracini Cunha, Isabella Pompeu Mattos Almeida, Andrew Oliveira Silva, Náthali Silva Paiva, Carmen Regla Vargas, Adriana Fernanda Kuckartz Vizuete, Márcia Giovenardi, Alethéa Gatto Barschak

**Affiliations:** 1 Programa de Pós-Graduação em Ciências da Saúde, Universidade Federal de Ciências da Saúde de Porto Alegre (UFCSPA), Avenida Sarmento Leite 245, Porto Alegre 90050-170, Brazil; luizaferracini@gmail.com (L.F.C.); marciag@ufcspa.edu.br (M.G.); 2 Laboratório de Análises Clínicas, Universidade Federal de Ciências da Saúde de Porto Alegre (UFCSPA), Avenida Sarmento Leite 245, Porto Alegre 90050-170, Brazil; isabella.almeida@ufcspa.edu.br; 3 Laboratório de Células, Tecidos e Genes, Centro de Pesquisas Experimentais, Hospital de Clínicas de Porto Alegre, Rua Ramiro Barcelos 2350, Porto Alegre 90035-903, Brazil; andrewosilva@hcpa.edu.br (A.O.S.); nspaiva@hcpa.edu.br (N.S.P.); 4 Serviço de Genética Médica, Hospital de Clínicas de Porto Alegre, Rua Ramiro Barcelos 2350, Porto Alegre 90035-903, Brazil; rvargas@hcpa.edu.br; 5 Departamento de Ciências Básicas da Saúde, Universidade Federal de Ciências da Saúde de Porto Alegre (UFCSPA), Avenida Sarmento Leite 245, Porto Alegre 90050-170, Brazil; adriana.vizuete@ufcspa.edu.br

**Keywords:** high-protein diet, probiotics, oxidative stress, mitochondrial function, lipid profile, amino acid profile

## Abstract

Background/Objectives: High-protein (HP) diets may influence body composition, lipid metabolism, oxidative stress, and mitochondrial function, but their interaction with probiotics remains unclear. This study evaluated the effects of HP feeding, alone or combined with *Lactobacillus acidophilus* and *Bifidobacterium lactis*, on metabolic, redox, mitochondrial, and plasma amino acid parameters in Wistar rats. Methods: Thirty-two male Wistar rats were allocated to standard diet (SD), SD plus probiotics, HP diet, or HP plus probiotics for 10 weeks. Body weight gain, food and energy intake, visceral adiposity, plasma biochemical profile, adipose tissue histology, oxidative stress markers, mitochondrial electron transport chain activity, and plasma amino acids were assessed. Results: Food and energy intake did not differ among groups. HP-fed animals showed lower weight gain, reduced visceral fat, and lower total cholesterol and triglycerides. Probiotics modulated selected redox-related outcomes, including methylglyoxal and sulfhydryl levels, with tissue-specific responses. Complex I activity increased in adipose tissue and skeletal muscle, and plasma amino acid profiles were altered, particularly glutamate, leucine, aspartate, alanine, and methionine. Conclusions: HP feeding, with or without probiotics, modulated metabolic, redox, mitochondrial, and amino acid-related parameters in Wistar rats. These findings suggest that HP diets and probiotics may influence cardiometabolic adaptations, although tissue-specific responses and mechanisms require further investigation.

## 1. Introduction

Obesity and related metabolic disorders, including dyslipidemia, insulin resistance, and cardiovascular diseases, remain major public health concerns worldwide. These conditions are frequently associated with excessive caloric intake, impaired energy balance, and visceral adipose tissue accumulation, which increase cardiometabolic risk [1]. Thus, dietary interventions are central strategies for body weight management and metabolic improvement [2].

High-protein diets have gained attention as nutritional strategies for weight control and cardiometabolic modulation. Higher protein intake may promote satiety, increase diet-induced thermogenesis, and preserve lean body mass during caloric restriction [3,4]. Protein-rich diets have also been associated with improvements in lipid metabolism, including reductions in total cholesterol, triglycerides, and very-low-density lipoprotein levels [5]. Beyond these effects, high-protein diets may influence oxidative stress, a process involved in metabolic disorders and characterized by an imbalance between reactive oxygen species production and antioxidant defenses [6,7]. However, their effects on antioxidant capacity and redox-sensitive molecules, such as sulfhydryl groups, remain under investigation [8].

Mitochondrial function is closely linked to metabolic health, as mitochondria are the primary sites of oxidative phosphorylation and energy production [9]. Dietary components, including proteins and amino acids, can influence mitochondrial bioenergetics, while electron transport chain activity in adipose and skeletal muscle tissues reflects tissue-specific adaptations to nutritional interventions [10,11].

Probiotic supplementation has also emerged as a strategy for improving metabolic health in obesity-related disorders. Probiotics are live microorganisms that, when administered in adequate amounts, may confer health benefits through multiple mechanisms involving the intestinal environment, barrier function, immune signaling, and host metabolism [12]. Evidence suggests that probiotics may improve intestinal barrier function, reduce systemic inflammation, influence lipid metabolism, enhance insulin sensitivity, and mitigate oxidative stress [13]. Their interaction with high-protein diets is therefore relevant, as probiotics may modulate systemic metabolic and redox responses to dietary interventions [14].

Given that high-protein feeding increases the delivery of protein-derived substrates to the gastrointestinal tract and may modify host metabolic signaling, probiotic strains capable of modulating intestinal barrier function, oxidative balance, and lipid metabolism may influence the systemic response to increased protein intake [14,15,16]. *L. acidophilus* and *B. lactis* were selected based on their reported antioxidant, anti-inflammatory, and gut-related effects [15,16]. We hypothesized that supplementation with these strains would modulate the metabolic, redox, and mitochondrial adaptations induced by high-protein feeding. However, their interaction with high-protein diets remains insufficiently understood. Therefore, this study evaluated the effects of a high-protein diet, alone or combined with *Lactobacillus acidophilus* and *Bifidobacterium lactis*, on body composition, lipid profile, oxidative stress, mitochondrial function, and plasma amino acid levels in Wistar rats.

## 2. Materials and Methods

### 2.1. Animals, Diets, and Experimental Design

This study followed ARRIVE 2.0, CONCEA (Brazil), and the Guide for the Care and Use of Laboratory Animals. The experimental protocol was approved by the Ethics Committee on the Use of Animals (CEUA) of the Universidade Federal de Ciências da Saúde de Porto Alegre (UFCSPA) (protocol No. 355/2023; approved on 28 April 2023). Thirty-two male Wistar rats (~8 weeks old, ±350 g) were included in the study. Animals were housed in collective cages (*n* = 2) at 20–24 °C under a 12:12 h light-dark cycle, with water ad libitum. The intervention started at 8 weeks of age and lasted 10 weeks. No inclusion or exclusion criteria were applied, no exclusions occurred, and no specific strategies were used to minimize potential confounders. No formal a priori power calculation was performed. The sample size was prospectively defined as eight animals per group based on feasibility and animal availability for the planned experimental procedures.

Animals were allocated into four groups: standard diet (SD, 26% energy from protein), standard diet plus probiotics (SDP, 26% energy from protein), high-protein diet (HP, 52.7% energy from protein), and high-protein diet plus probiotics (HPP, 52.7% energy from protein). Nutritional specifications are shown in Table 1.

The primary outcomes were body weight gain and visceral adipose tissue accumulation; secondary outcomes included lipid profile, oxidative stress markers, mitochondrial function, and plasma amino acid levels.

### 2.2. Probiotic Supplementation

*Lactobacillus acidophilus* (1 × 10^9^ CFU/mL) and *Bifidobacterium lactis* (1 × 10^9^ CFU/mL) were combined in an aqueous formulation prepared by a compounding pharmacy. A volume of 1 mL was administered by oral gavage three times weekly for 10 weeks to the SDP and HPP groups, corresponding to a nominal dose of 1 × 10^9^ CFU of each microorganism per administration. The SD and HP groups received 1 mL of water as vehicle. Viable CFU counts were not independently monitored by the research team during the intervention. Because the formulation was aqueous, new preparations were obtained every three weeks according to the stability period specified by the compounding pharmacy, in order to minimize potential loss of viability.

### 2.3. Body Weight, Lee Index, Food Intake, and Energy Consumption

Animals were weighed weekly, and naso-anal length was measured at the end of the experiment for Lee Index calculation.

Food intake was recorded weekly at the cage level, with two animals housed per cage. The amount of food consumed by each cage was divided by two to estimate the mean food intake per animal and was converted into energy intake (kcal) according to the caloric density of each diet. Because food consumption was measured at the cage level, the cage was considered the experimental unit for food and energy intake analyses (*n* = 4 cages per group).

### 2.4. Tissue Collection

At the end of treatment, animals were fasted for 4 h and euthanized by decapitation without anesthesia, as approved by the UFCSPA Ethics Committee on the Use of Animals (CEUA; protocol No. 355/2023). Pre-euthanasia anesthetic agents were not used because general anesthetics may directly interfere with mitochondrial respiration and electron transport chain activity [17]. Blood, visceral adipose tissue, including epididymal and mesenteric depots, and skeletal muscle tissue (rectus femoris) were collected and stored at −80 °C for further analysis or fixed in formalin for histological analysis. All fat depots were dissected by an experienced researcher to prevent cross-contamination with other tissues.

### 2.5. Biochemical Profile and Methylglyoxal Assessment

Plasma glucose, total cholesterol, HDL-cholesterol, and triacylglycerol concentrations were measured using commercial kits according to the manufacturer’s instructions (Bioclin/Quibasa © 2012, Belo Horizonte, Brazil).

Plasma methylglyoxal (MG) was estimated using a glyoxalase 1 (GLO1)-based spectrophotometric assay according to Racker [18], with minor modifications, as previously described by Vizuete and Gonçalves [19]. A five-point MG calibration curve spanning 0.078–1.25 mM was used. For each sample, 25 µL of plasma was added to a final reaction volume of 0.25 mL containing 100 mM KH_2_PO_4_ buffer (adjusted to pH 6.6 with 1 M KOH), 2 mM reduced glutathione freshly prepared in sodium bicarbonate solution (pH 6.6), and 3.14 µL (1 U) glyoxalase I in UV-transparent 96-well plates. MG reacts non-enzymatically with reduced glutathione to form a hemithioacetal, which is subsequently converted by GLO1 to S-D-lactoylglutathione, a physiological intermediate of the glutathione-dependent glyoxalase pathway and the substrate of glyoxalase 2. S-D-lactoylglutathione exhibits strong absorption in the low-ultraviolet range, and its formation can therefore be assessed spectrophotometrically at 240 nm [20]. After incubation for 30 min at 37 °C, absorbance was measured at 240 nm. A blank measurement performed under the assay conditions was subtracted from the sample readings. Plasma MG concentrations were estimated from the calibration curve and expressed as µmol/L.

### 2.6. Histological Analysis

For adipose tissue morphometric analysis, micrographs from three independent fields of the histological section were obtained for each animal using a 20× objective and light microscopy (BX-61, Olympus Corporation, Tokyo, Japan). Images were processed using Fiji software 2.9.0 (National Institutes of Health, Bethesda, MD, USA) [21] to improve visualization of adipocyte boundaries, and adipocyte area was measured using Image Pro Plus 6.0 (Media Cybernetics, Silver Spring, MD, USA) by an investigator blinded to the experimental groups. Approximately 180 adipocytes were evaluated per micrograph, corresponding to approximately 540 cells per animal across the three analyzed fields. All adipocytes entirely contained within the image frame and meeting the predefined morphometric criteria were included; therefore, no individual cell subsampling according to adipocyte size was performed. Adipocytes touching the image borders were not included, and objects with an area below 350 µm^2^ were excluded to minimize the inclusion of stromal or non-adipocyte structures [22]. The physical area of each micrograph was calibrated from the image scale (200 µm corresponding to 450 pixels), resulting in an analyzed field area of 0.63766 mm^2^. Adipocyte counts were normalized to the analyzed field area and expressed as cells/mm^2^. For each animal, adipocyte area and adipocyte count per analyzed area were averaged across the three fields, and the animal-level values were used for group comparisons.

### 2.7. Redox Profile Assessment

Adipose and skeletal muscle tissues were homogenized in sodium phosphate buffer (20 mM, pH 7.4) containing 140 mM KCl and centrifuged at 3500 rpm for 10 min at 4 °C. Supernatants were stored at −80 °C until analysis. Thiobarbituric acid reactive substances (TBARS) were measured according to Ohkawa et al. [23], using 1,1,3,3-tetramethoxypropane for calibration, and expressed as nmol MDA/mg protein. Carbonyls were determined spectrophotometrically according to Reznick and Packer [24] and expressed as nmol carbonyl groups/mg protein. Sulfhydryl levels were measured by DTNB reduction at 412 nm [25] and expressed as nmol TNB/mg protein. Superoxide dismutase (SOD) activity was determined by inhibition of adrenaline auto-oxidation at 480 nm according to Misra and Fridovich [26] and expressed as U SOD/mg protein.

### 2.8. Assessment of Mitochondrial Electron Transport Chain Activity

For ETC complex determination, adipose and skeletal muscle tissues were homogenized (1:20, *w*/*v*) in 250 mM sucrose buffer and centrifuged at 3500 rpm for 10 min at 4 °C. Supernatants were stored frozen, and samples underwent three freeze–thaw cycles to disrupt mitochondrial membranes and expose ETC enzymes. Complexes I, II, III, and IV activities were determined according to Spinazzi et al. [27] and expressed as nmol/min/mg protein.

### 2.9. Protein Quantification

Proteins in adipose and skeletal muscle tissue homogenates were determined according to Lowry and collaborators [28] using bovine serum albumin as a standard.

### 2.10. Plasma Amino Acid Analysis

The free amino acids in plasma were determined by reversed-phase HPLC according to Joseph and Marsden [29].

### 2.11. Statistical Analysis

Statistical analyses were conducted using GraphPad Prism 9 (GraphPad Software, San Diego, CA, USA). Data are presented as means ± SD. The animal was considered the experimental unit for individual-level outcomes (*n* = 8 per group unless otherwise specified; plasma amino acid analysis: *n* = 6 per group). For food and energy intake, which were measured at the cage level, the cage was considered the experimental unit (*n* = 4 cages per group). Differences were examined using two-way analysis of variance (ANOVA) followed by Bonferroni post hoc comparisons. Values of *p* < 0.05 were considered statistically significant.

## 3. Results

### 3.1. Food Intake, Body Weight, and Visceral Adiposity

**Figure 1:**
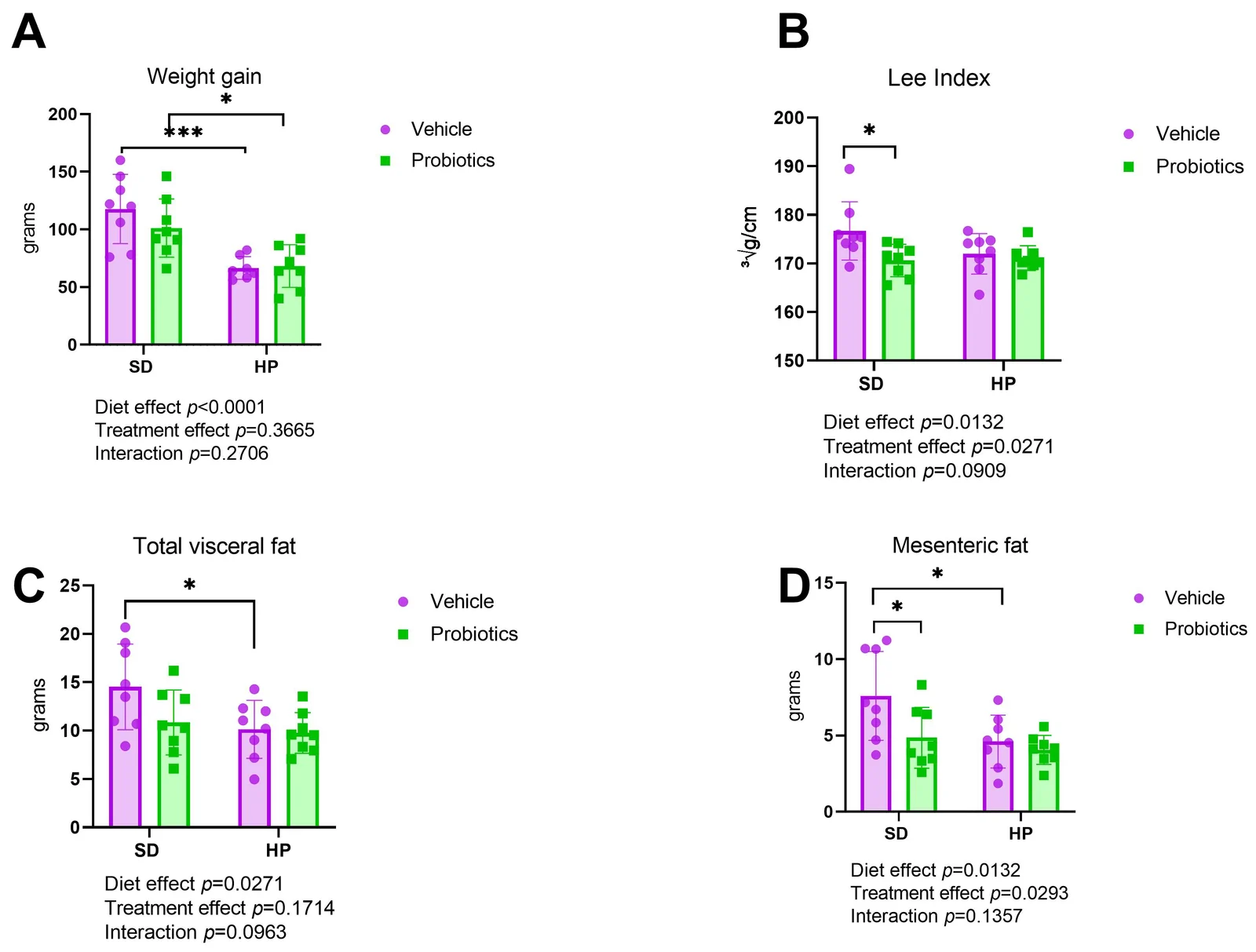
(**A**) Weight gain; (**B**) Lee index; (**C**) total visceral fat; (**D**) mesenteric fat. Values are mean ± SD (*n* = 8). Symbols indicate Bonferroni post hoc comparisons: * *p* < 0.05, *** *p* < 0.001. SD, standard diet; HP, high-protein diet.

Food and energy intake did not differ among groups. HP animals gained less weight than SD (*p* = 0.0003), and HPP gained less than SDP (*p* = 0.0144; Figure 1A). Lee index was lower in SDP than in SD (*p* = 0.0148; Figure 1B). Total visceral fat was lower in HP than in SD (*p* = 0.0271), and total mesenteric fat was reduced in SDP (*p* = 0.0227) and HP (*p* = 0.0125) compared with SD; epididymal fat did not differ among groups (Figure 1).

### 3.2. Plasma Biochemical Profile and Methylglyoxal

Biochemical analysis showed no differences in glucose levels (Table 2). HP feeding reduced total cholesterol in HP versus SD (*p* = 0.0410) and HPP versus SDP (*p* = 0.0187). HDL showed a diet effect (*p* = 0.0085), triglycerides were lower in HP versus SD and HPP versus SDP (*p* < 0.0001 for both), and plasma methylglyoxal showed a significant diet × probiotic interaction (*p* = 0.0096). MG concentrations were lower in SDP compared with SD (*p* < 0.0001) and in HP compared with SD (*p* = 0.0007), whereas no additional significant reduction was observed in HPP compared with HP (Table 2). 

### 3.3. Adipose Tissue Histology and Redox Profile

**Figure 2:**
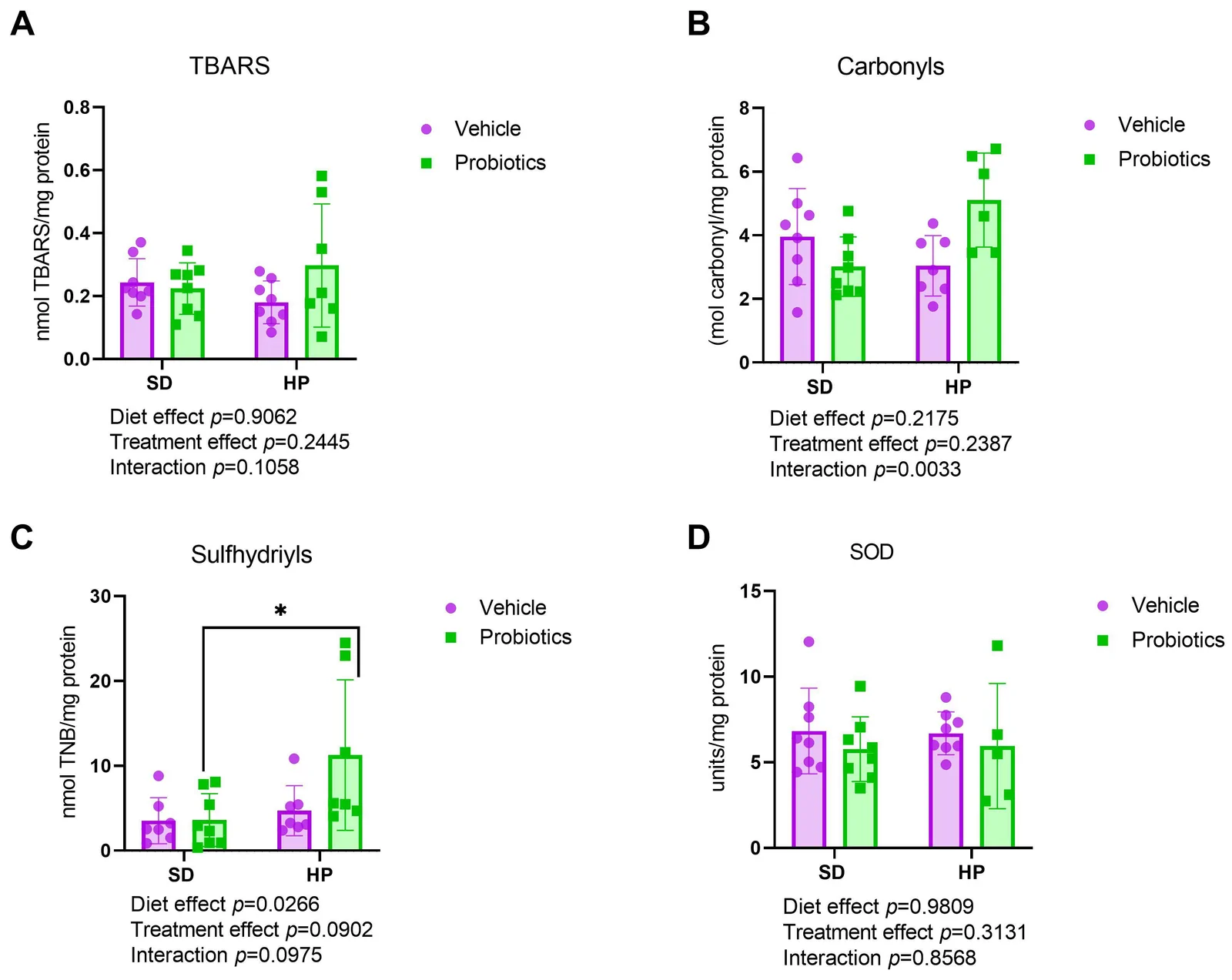
Redox markers in adipose tissue: (**A**) TBARS; (**B**) carbonyls; (**C**) sulfhydryl levels; (**D**) SOD activity. Values are mean ± SD (*n* = 8). Symbols indicate Bonferroni post hoc comparisons: * *p* < 0.05. TBARS, thiobarbituric acid reactive substances; SOD, superoxide dismutase; SD, standard diet; HP, high-protein diet.

Adipose tissue histology showed no significant differences in adipocyte area or adipocyte count per analyzed area among groups (Table 3). In adipose tissue, sulfhydryl levels were higher in the HPP group than in the SDP (*p* = 0.0266) group, whereas TBARS, carbonyl, and SOD levels did not differ among groups (Figure 2).

### 3.4. Skeletal Muscle Redox Profile

**Figure 3:**
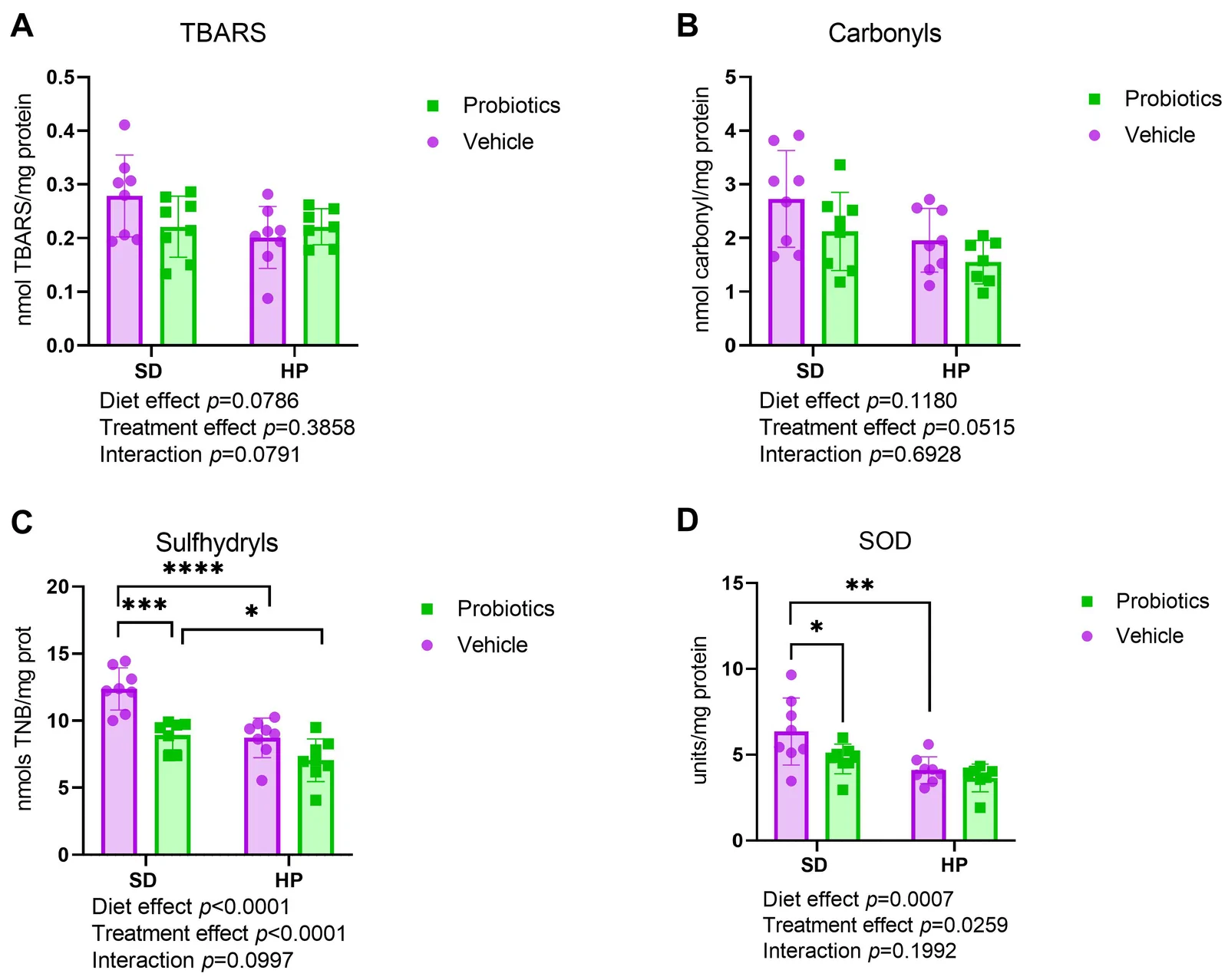
Redox markers in skeletal muscle: (**A**) TBARS; (**B**) carbonyls; (**C**) sulfhydryl levels; (**D**) SOD activity. Values are mean ± SD (*n* = 8). Symbols indicate Bonferroni post hoc comparisons: * *p* < 0.05, ** *p* < 0.01, *** *p* < 0.001, **** *p* < 0.0001. TBARS, thiobarbituric acid reactive substances; SOD, superoxide dismutase; SD, standard diet; HP, high-protein diet.

In skeletal muscle, sulfhydryl levels were lower in the SDP (*p* = 0.0002) and HP (*p* = 0.0019) groups compared with the SD group and were also lower in HPP than in SDP. SOD activity was reduced in the SDP (*p* = 0.0007) and HP (*p* = 0.0259) groups compared with SD, while TBARS and carbonyl levels remained unchanged (Figure 3).

### 3.5. Mitochondrial Electron Transport Chain Activity

**Figure 4:**
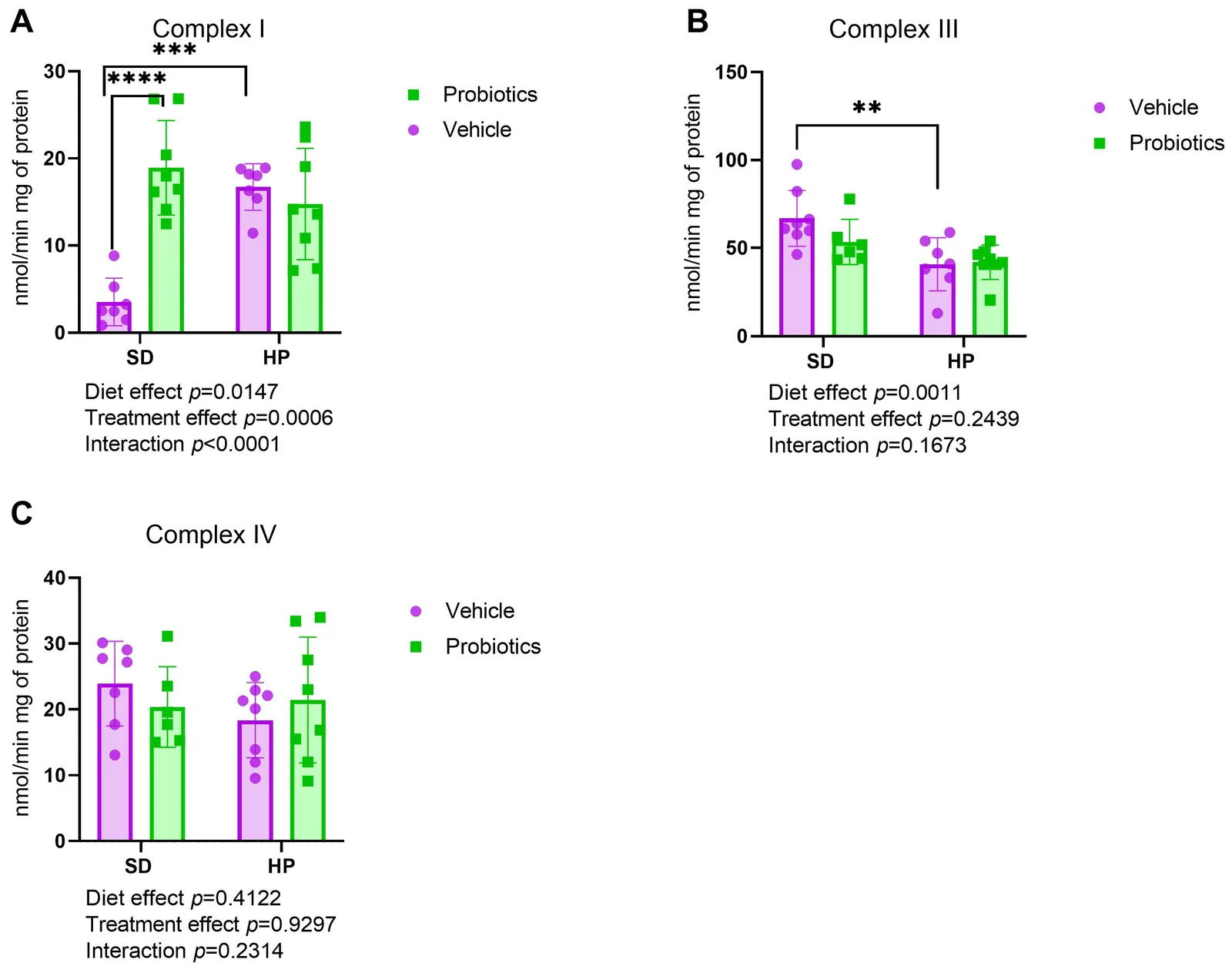
Electron transport chain activity in adipose tissue: (**A**) Complex I; (**B**) Complex III; (**C**) Complex IV. Values are mean ± SD (*n* = 8). Symbols indicate Bonferroni post hoc comparisons: ** *p* < 0.01, *** *p* < 0.001, **** *p* < 0.0001. SD, standard diet; HP, high-protein diet.

**Figure 5:**
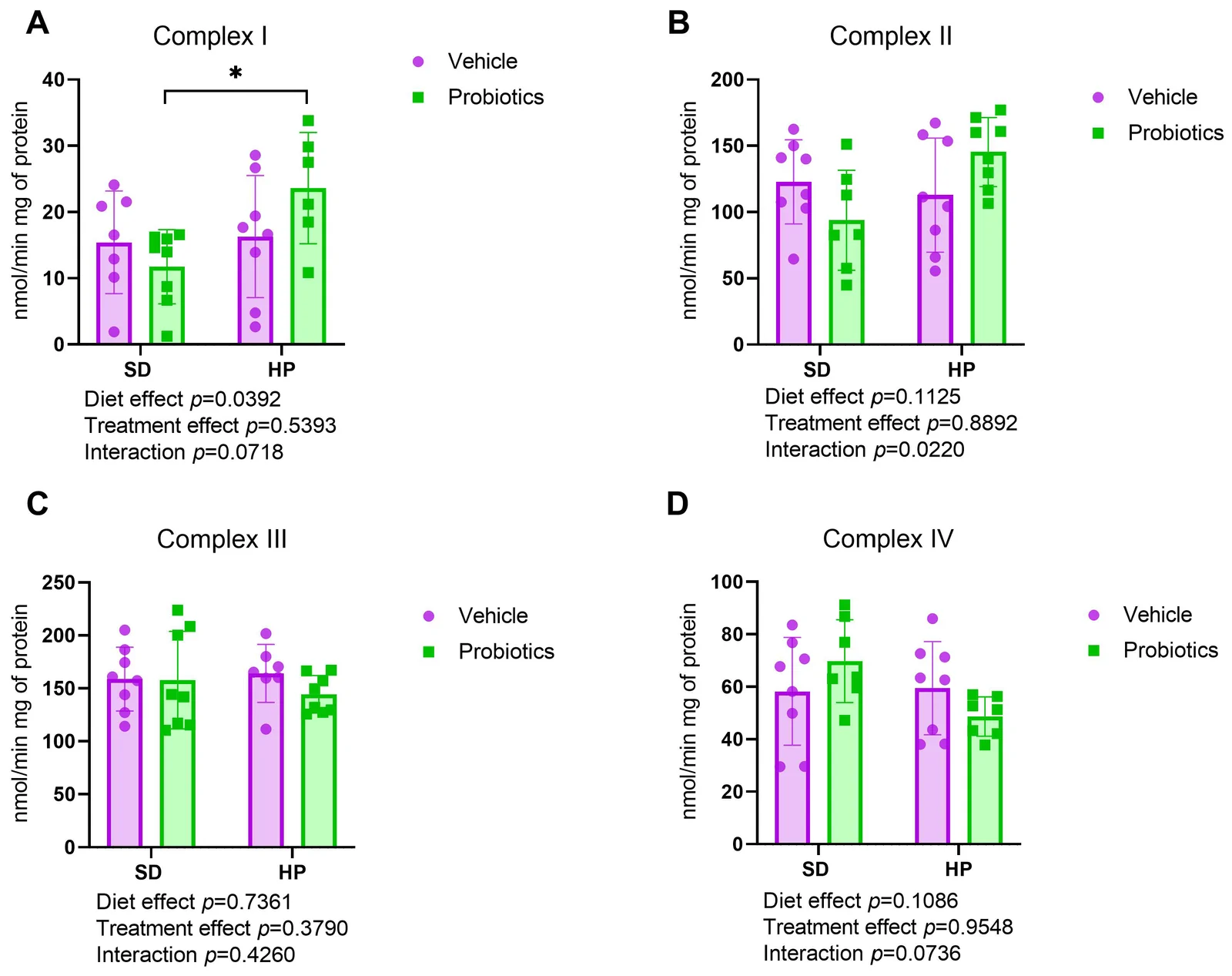
Electron transport chain activity in skeletal muscle: (**A**) Complex I; (**B**) Complex II; (**C**) Complex III; (**D**) Complex IV. Values are mean ± SD (*n* = 8). Symbols indicate Bonferroni post hoc comparisons: * *p* < 0.05. SD, standard diet; HP, high-protein diet.

In adipose tissue, mitochondrial Complex I activity was increased in the SDP (*p* < 0.0001) and HP (*p* < 0.001) groups compared with SD, whereas Complex III activity was lower in HP (*p* = 0.0021) than in SD; Complex IV did not differ among groups (Figure 4). In skeletal muscle, Complex I activity was higher in the HP group (*p* = 0.019) compared with SD, with no significant differences in Complexes II, III, or IV (Figure 5).

### 3.6. Plasma Amino Acid Profile

Plasma amino acid analysis showed lower glutamate levels in HP versus SD (*p* = 0.0253) and in HPP versus SDP (*p* = 0.0112), whereas leucine was higher in HP compared with SD (*p* = 0.0380). Aspartate and alanine were lower in HPP compared with SDP (*p* = 0.0073 and *p* = 0.0277, respectively). Methionine was lower in HP versus SD (*p* = 0.0114) and in HPP versus SDP (*p* = 0.0046). No significant differences were observed for the other amino acids analyzed (Table 4).

## 4. Discussion

This study evaluated the effects of a high-protein (HP) diet, with or without probiotic supplementation, on weight gain, lipid metabolism, body composition, oxidative stress, mitochondrial function, and plasma amino acid profile in Wistar rats. Overall, HP feeding was associated with lower weight gain, reduced visceral adiposity, improved lipid parameters, and tissue-specific redox and mitochondrial adaptations, while probiotics modulated selected metabolic and oxidative stress-related outcomes.

The lower weight gain observed in the HP groups supports the role of high-protein diets in modulating energy balance and reducing fat accumulation, as previously described by Pesta and Samuel [30]. Since food and caloric intake did not differ among groups, these effects were not solely explained by reduced energy intake, but may involve increased diet-induced thermogenesis, enhanced satiety, and improved energy expenditure [3,4]. These findings are consistent with evidence that dietary protein influences appetite regulation and energy metabolism [31]. Although caloric intake was similar in this experimental setting, HP diets are often associated with lower spontaneous energy intake through satiety-related mechanisms involving GLP-1 and PYY [31,32]. The higher metabolic cost of protein digestion, absorption, and amino acid processing may also increase diet-induced thermogenesis and contribute to lower fat accumulation without explicit caloric restriction [33,34,35]. Importantly, the experimental HP diet differed from the standard diet not only in protein content but also in carbohydrate proportion (37.5% vs. 63.0% of energy) and energy density (3.74 vs. 4.26 kcal/g). Although calculated energy intake did not differ significantly among groups, these compositional differences may have independently contributed to the observed metabolic responses. Accordingly, the present findings should be interpreted as effects of the complete high-protein dietary formulation rather than of protein enrichment in isolation. The complete fiber and non-digestible fraction of the two diets was not independently quantified; therefore, potential differences in gastrointestinal bulk, fermentable substrate availability, or transit cannot be excluded.

The lower Lee index in the SDP group suggests that probiotics may influence body composition, particularly metabolically active visceral and mesenteric fat depots [36]. This is relevant because visceral adiposity is associated with insulin resistance, systemic inflammation, and cardiovascular risk [37,38]. The absence of changes in epididymal fat and adipocyte morphology suggests that the interventions promoted systemic metabolic adaptations rather than localized adipose remodeling alone, reinforcing that adipose depots may respond differently to dietary interventions [39].

The reductions in total cholesterol and triglycerides in HP-fed groups indicate favorable effects on lipid metabolism. High-protein intake may support lipid oxidation and reduce hepatic triglyceride synthesis [40], while HP diets may also reduce VLDL secretion and modulate lipoprotein metabolism, particularly with lower carbohydrate availability [41,42]. Stable glucose levels alongside changes in cholesterol and methylglyoxal (MG) suggest metabolic adaptations independent of glycemic changes.

Lower MG levels in probiotic-treated groups may reflect protection against MG-related oxidative and inflammatory damage, possibly linked to the effects of probiotics on gut health, lipid metabolism, and oxidative stress regulation [43]. Probiotics may also support antioxidant defenses and glyoxalase-related pathways, reducing MG accumulation and advanced glycation end-product formation, both associated with chronic inflammation and cardiometabolic dysfunction [44].

Redox findings indicate tissue-specific responses to HP feeding and probiotics. In adipose tissue, higher sulfhydryl levels in the HPP group suggest increased antioxidant defenses in response to the oxidative demands of protein metabolism. Sulfhydryl groups protect against protein oxidation and may help preserve redox balance under metabolic load [45,46]. In skeletal muscle, reduced sulfhydryl levels and lower SOD activity suggest a more limited antioxidant response, indicating that adipose tissue and skeletal muscle differ in adaptive capacity to HP diets and probiotics [47]. Because SOD converts superoxide radicals into less reactive species, reduced SOD activity may reflect impaired muscle redox regulation [26,47]. These findings are consistent with experimental evidence showing that high-protein diets can modify oxidative status across different tissues and experimental contexts [8,48,49].

The opposite sulfhydryl responses observed in adipose tissue and skeletal muscle may reflect tissue-specific differences in thiol redox buffering, substrate utilization, mitochondrial activity, and antioxidant enzyme capacity [45,46,50]. Adipose tissue and skeletal muscle have distinct metabolic roles and may therefore respond differently to increased amino acid availability and probiotic-associated metabolic signals. However, because GSH/GSSG balance and the signaling pathways regulating thiol homeostasis were not directly assessed, the mechanisms underlying these divergent responses cannot be established from the present data.

Regarding mitochondrial function, increased Complex I activity in adipose tissue and skeletal muscle indicates an adaptation in electron transport chain enzymatic activity following HP feeding and/or probiotic supplementation. These findings are consistent with the ability of dietary interventions to modify mitochondrial enzyme activity in metabolically active tissues [9,50]. However, because the analyses were performed in tissue homogenates and did not assess integrated mitochondrial respiration or ATP production, increased Complex I activity should not be interpreted as direct evidence of enhanced mitochondrial oxidative capacity.

The plasma amino acid profile further supports systemic metabolic adaptation, with changes in glutamate, leucine, aspartate, alanine, and methionine. Increased leucine is relevant because this branched-chain amino acid is involved in mTOR signaling, protein synthesis, and energy metabolism [51]. Methionine participates in methylation and redox balance, including glutathione-related pathways, which may help explain the higher adipose sulfhydryl content in the HPP group. Together, these changes suggest that HP feeding, particularly with probiotics, may modulate pathways related to oxidative stress, energy homeostasis, and muscle metabolism [51,52].

This study has limitations. The sample size and intervention period may limit generalizability and long-term interpretation. The use of an animal model restricts direct extrapolation to humans. Physical activity was not assessed, although all groups were maintained under the same experimental conditions, likely minimizing differences in spontaneous activity. Further studies including longer interventions, mechanistic analyses, and microbiota-related outcomes are needed to clarify the observed metabolic, redox, and mitochondrial effects. An additional limitation is the exclusive use of male rats. Sex-related differences in adipose tissue distribution, substrate metabolism, mitochondrial function, and redox regulation may result in distinct responses to high-protein diets and probiotic supplementation. Therefore, the present findings should not be directly extrapolated to female animals, and future studies should evaluate both sexes.

## 5. Conclusions

High-protein feeding, with or without probiotics, modulated body weight gain, visceral adiposity, lipid profile, redox status, mitochondrial function, and plasma amino acid levels in Wistar rats. These findings suggest potential cardiometabolic adaptations to high-protein diets and probiotics, although tissue-specific responses and underlying mechanisms require further investigation.

## Figures and Tables

**Table 1:** Diet composition as a percentage of total energy.

Component	Standard Diet	High-Protein Diet
Protein (% kcal)	26.0	52.7
Carbohydrates (% kcal)	63.0	37.5
Fat (% kcal)	11.0	9.8
Energy density (kcal/g)	4.26	3.74

The high-protein diet was produced by Pragsoluções Biociências (Jaú, São Paulo, Brazil), and the standard diet (NuviLab^®^—Colombo, Paraná, Brazil) was supplied by UFCSPA as pellets.

**Table 2:** Plasma biochemical profile of Wistar rats fed a standard or high-protein diet, with or without probiotics.

Parameters	Standard Diet		High-Protein Diet		Two-Way ANOVA		
	Vehicle (SD)	Probiotics (SDP)	Vehicle (HP)	Probiotics (HPP)	Diet Effect	Treatment Effect	Interaction
Glucose (mg/dL)	152.1 ± 41.8	162.1 ± 25.0	145.1 ± 29.3	153.4 ± 21.9	0.4702	0.4035	0.9357
Triglycerides (mg/dL)	106.3 ± 30.5 ^a^	96.3 ± 36.4 ^b^	35.4 ± 6.5 ^a^	35.3 ± 8.5 ^b^	<0.0001	0.2700	0.9937
Total cholesterol (mg/dL)	60.6 ± 12.9 ^c^	59.5 ± 17.4 ^d^	46.0 ± 6.9 ^c^	43.0 ± 6.7 ^d^	0.0009	0.6385	0.8140
HDL (mg/dL)	23.4 ± 2.4	21.8 ± 4.5	19.3 ± 4.4	18.5 ± 2.3	0.0085	0.3509	0.7335
Methylglyoxal (µmol/L)	71.2 ± 6.4 ^e,f^	47.9 ± 14.1 ^e^	53.3 ± 5.0 ^f^	47.3 ± 6.5	0.0059	<0.0001	0.0096

Values are expressed as mean ± SD (*n* = 8). Matching lowercase superscript letters within each row identify pairs of groups showing a statistically significant difference in Bonferroni post hoc comparisons (*p* < 0.05, two-way ANOVA followed by Bonferroni post hoc test). SD, standard diet; SDP, standard diet plus probiotics; HP, high-protein diet; HPP, high-protein diet plus probiotics.

**Table 3:** Histological analysis of adipose tissue.

Parameters	Standard Diet		High-Protein Diet		Two-Way ANOVA		
	Vehicle (SD)	Probiotics (SDP)	Vehicle (HP)	Probiotics (HPP)	Diet Effect	Treatment Effect	Interaction
Adipocyte area (μm^2^)	2513.0 ± 474.1	2461.4 ± 427.9	2510.6 ± 574.1	2957.5 ± 316.7	0.1491	0.2447	0.1454
Adipocyte count per analyzed area(cells/mm^2^)	275.9 ± 45.4	294.1 ± 46.3	309.8 ± 61.7	262.5 ± 25.8	0.9471	0.3954	0.0621

Values are expressed as mean ± SD (*n* = 8). Two-way ANOVA followed by Bonferroni post hoc test. SD, standard diet; SDP, standard diet plus probiotics; HP, high-protein diet; HPP, high-protein diet plus probiotics.

**Table 4:** Plasma amino acid concentrations of Wistar rats fed a standard or high-protein diet, with or without probiotics.

Parameters (µmol/L)	Standard Diet		High-Protein Diet		Two-Way ANOVA		
	Vehicle (SD)	Probiotics (SDP)	Vehicle (HP)	Probiotics (HPP)	Diet Effect	Treatment Effect	Interaction
Glutamate	84.1 ± 16.1 ^a^	85.5 ± 24.4 ^b^	56.8 ± 14.7 ^a^	54.5 ± 11.4 ^b^	0.0005	0.9490	0.7991
Serine	179.7 ± 25.2	176.0 ± 25.5	154.4 ± 34.7	151.8 ± 29.6	0.1025	0.9093	0.7432
Tryptophan	100.3 ± 13.4	87.0 ± 25.2	79.9 ± 17.6	67.3 ± 18.3	0.0181	0.1127	0.9680
Valine	161.0 ± 25.3	162.2 ± 23.2	214.0 ± 55.7	163.6 ± 50.4	0.1223	0.1600	0.1423
Leucine	117.2 ± 16.7 ^c^	120.2 ± 16.6	162.8 ± 41.9 ^c^	128.1 ± 39.1	0.0419	0.2245	0.1516
Isoleucine	73.0 ± 13.4	74.0 ± 10.0	102.2 ± 26.1	75.8 ± 24.5	0.0687	0.1300	0.1060
Aspartate	5.7± 1.5	6.3 ± 1.4 ^d^	4.2 ± 0.6	3.8 ± 1.4 ^d^	0.0012	0.8122	0.3878
Glutamine	701.5 ± 56.6	609.6 ± 117.0	765.3 ± 146.6	764.0 ± 160.9	0.0478	0.3789	0.3918
Arginine	93.3 ± 7.5	85.8 ± 20.2	102.1 ± 24.4	82.2 ± 26.6	0.7638	0.1264	0.4761
Alanine	384.4 ± 33.5	376.2 ± 75.1 ^e^	344.2 ± 78.6	270.8 ± 73.3 ^e^	0.0158	0.1551	0.2520
Tyrosine	70.3 ± 3.1	69.7 ± 12.6	68.0 ± 5.2	56.0 ± 15.1	0.0724	0.1473	0.1875
Methionine	50.2 ± 4.6 ^f^	49.2 ± 7.4 ^g^	38.6 ± 6.3 ^f^	36.2 ± 7.2 ^g^	0.0002	0.5154	0.7842
Phenylalanine	57.4 ± 5.8	56.2 ± 8.8	59.5 ± 11.4	53.6 ± 12.8	0.9473	0.3920	0.5747
Lysine	263.9 ± 21.5	262.1 ± 54.5	256.0 ± 55.6	226.4 ± 47.3	0.2666	0.4190	0.4743
Ornithine	38.6 ± 21.4	36.0 ± 5.0	34.3 ± 11.3	29.6 ± 10.5	0.3362	0.5149	0.8500

Values are expressed as mean ± SD (*n* = 6). Matching lowercase superscript letters within each row identify pairs of groups showing a statistically significant difference in Bonferroni post hoc comparisons (*p* < 0.05, two-way ANOVA followed by Bonferroni post hoc test). SD, standard diet; SDP, standard diet plus probiotics; HP, high-protein diet; HPP, high-protein diet plus probiotics.

## Data Availability

The data presented in this study are available on request from the corresponding author. The data are not publicly available due to ongoing analyses and planned publications.

## Abbreviations

ANOVA	analysis of variance
CFU	colony-forming units
DTNB	5,5′-dithiobis-(2-nitrobenzoic acid)
ETC	electron transport chain
GLO1	glyoxalase 1
HDL	high-density lipoprotein
HP	high-protein diet
HPLC	high-performance liquid chromatography
HPP	high-protein diet plus probiotics
MDA	malondialdehyde
MG	methylglyoxal
SD	standard diet
SDP	standard diet plus probiotics
SOD	superoxide dismutase
TBARS	thiobarbituric acid reactive substances
TNB	2-nitro-5-thiobenzoate
VLDL	very-low-density lipoprotein
